# An Observational Cross-Sectional Study on Cancer Awareness and Beliefs about Carcinogens among Saudi Women

**DOI:** 10.3390/ijerph19052528

**Published:** 2022-02-22

**Authors:** Khalid Akkour, Shahad Alsuwaidan, Mohammed Almoqren, Futoon Alsaleh, Lolowah Alghuson

**Affiliations:** 1 Obstetrics and Gynecology Department, College of Medicine, King Saud University, Riyadh 11461, Saudi Arabia; mzmoqren@gmail.com (M.A.); futoonsas@gmail.com (F.A.); anartist@live.com (L.A.); 2 Faculty of Medicine, College of Medicine, King Saud University, Riyadh 12371, Saudi Arabia; shahdalsowaidan@gmail.com

**Keywords:** carcinogens, cancer awareness, carcinogenic risk factors

## Abstract

Public knowledge about the risk factors of cancer is essential to ensure an effective prevention program. This study aims to explore the knowledge of the general Saudi population about cancer and carcinogens and to determine the misconceptions about carcinogens to help create appropriate evidence-based prevention awareness programs. A questionnaire of 63 questions related to biographic data, source of knowledge, risk factors, and the burden of cancer was distributed online. The internet was the most sought source for cancer-related information (75.2%). The inclusion of cancer-related subjects in the educational curriculum was believed to be the best way to educate children about cancer (48.4%). Knowledge about cancer risk factors was good overall for 10 out of the 27 risk factors assessed in the study, with cigarette smoking being the most well-known risk factor (91.5%), followed by hookah smoking (85.6%), and nuclear waste exposure (80%). However, only 16.3% of participants were aware of the risk associated with Oral Contraceptive Pills (OCPs), and less than half of the participants knew the risk associated with poor physical activity. About 45% (44.9%) believed that envy and spiritual causes are associated with cancer. Most of the study participants (70%) considered cancer to be a significant health issue in Saudi, mainly due to the increasing incidence rate (44%). Conclusion: This study showed a good level of awareness regarding cancer risk factors and environmental carcinogens, which could serve as a roadmap for future awareness programs and studies targeted at the knowledge of other risk factors for cancer.

## 1. Introduction

Cancer is a spectrum of diseases characterized by the uncontrolled proliferation of abnormal cells of any organ or tissue with the ability to invade adjacent and distant structures. According to World Health Organization (WHO), it is the second leading cause of death worldwide [1]. The international agency for research on cancer (IARC) states that globally, the number of new cases in 2020 reached 19,292,789, resulting in an estimated annual cost of USD 1.16 trillion, with death cases of 9,958,133 [2]. Among these numbers, the most prevalent types were breast cancer, lung cancer, prostate cancer, and colon cancer [2].

In Saudi Arabia, the incidence of different types of cancers has increased over the past decade [3]. In 2016, the number of reported new cases in the country reached 17,602 cases as breast, colorectal, and thyroid cancers accounted for the most cases in descending order [4].

A review paper published in 2019 indicated that new cancer cases in Saudi Arabia could reach 151,719 cases by 2025, and the number of deaths expected for the same year is estimated to be 30,718 [5]. Another correlating study conducted between 1999 and 2015 estimated that the number of cancer cases in the country has increased by 136% and is projected to increase by 63% by the year 2030 [6].

There are many known oncogenic factors, many of which are fortunately preventable. Most cancers are associated with modifiable and lifestyle-related risk factors such as cigarette smoking, alcohol consumption, physical inactivity, obesity, and dietary habits. Thus, cancer incidence can be reduced by following lifestyle-changing programs [7].

In the last 40 years, the socioeconomic situation in Saudi Arabia has improved drastically. People’s habits have evolved as a consequence of this social development and transition, shifting towards a sedentary lifestyle, processed food intake, and obesity [3], unaware of their association with cancer [8,9]. Another supporting study showed that 82.6% of 26,000 families surveyed across the 13 administrative regions of the country did not practice physical activity as stated by physical activity guidelines [9].

The current trends in the number of cases and the increasing prevalence of risk factors place a tremendous demand on health care resources and predict an even greater burden on health care systems in the future, both locally and globally. They also represent an opportunity for both the global health care systems and Saudi health authorities to implement preventative strategies and educative campaigns that consider the prevalent risk factors, of which 30–50% are preventable (WHO).The present study aimed at exploring the knowledge of the general Saudi population about cancer and carcinogens and determining the misconceptions about carcinogens in order to help create a culturally appropriate evidence-based prevention awareness program.

## 2. Materials and Methods

### 2.1. Study Design and Sample

This cross-sectional observational survey was conducted to evaluate Saudi women’s awareness of cancer and carcinogens. The study was approved by the King Saud University Institutional Review Board. Based on the Saudi female census of 2018, which was equivalent to 10,192,732 females, the sample size was estimated to be 385 (confidence level of 95% and a confidence interval of 5%). The sample was collected through convenient sampling.

### 2.2. Questionnaire

The questionnaire was first developed and validated by Ahmad et al. at King Hussein Institute for Biotechnology and Cancer (KHIBC) [10] It was reviewed by the knowledge, awareness, attitude survey advisory committee, the research team from the Center of Consultation (COC) at the University of Jordan and the Department of Statistics (DOS). The survey was then translated to Arabic using forward and back-translation, modified to the goals of this study and reviewed for consistency through a pilot of 50 participants. The questionnaire contains two sections: the first section collects sociodemographic data, and the second evaluates respondents’ awareness of carcinogens [10,11]. The questionnaire was then transformed into a self-administered electronic survey.

### 2.3. Data Collection and Recruitment

The electronic questionnaire was shared through social media platforms to reach female participants from all over the kingdom. Inclusion criteria involved all adult Saudi women. Exclusion criteria were non-Saudis, males, minors, and those who did not complete the survey. Participants filled out the online surveys anonymously and voluntarily without incentives, and each participant had a unique IP address to prevent duplications. Data collection started on the 1 January 2021 and concluded at the completion of the sample size on the 22 January 2021.

### 2.4. Data Analysis

Data were exported for analysis using the Statistical Package for the Social Sciences; SPSS statistics for Windows, version 21.0 (SPSS Inc., Chicago, IL, USA). Categorical variables were expressed as percentages. The level of awareness was assessed as a score out of 28, where a higher score represents a higher level of awareness. Ordinal logistic regression was then used to assess the factors associated with awareness levels regarding carcinogens. A *p*-value less than 0.05 was considered significant.

## 3. Results

**Figure 1:**
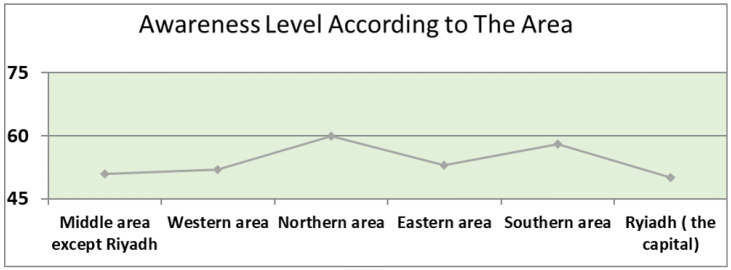
Awareness Level According to The Area.

Out of 459 participants, the vast majority lived in the Riyadh region, 68.8% (*n* = 316), followed by the Western region of Saudi Arabia, 13.1% (*n* = 60) (Figure 1). Moreover, 64.7% (*n* = 297) of participants were young (18–34 years old). Most participants were well educated with 71.2% (*n* = 327) having a bachelor’s degree. In addition, more than 90% (*n* = 416) were non-smokers and had never smoked a cigarette or hookah. The percentages of single and married females were nearly equal, at 49.0% (*n* = 225) and 47.3% (*n* = 217), respectively. More than 61% of participants were unemployed and dependent on their family income. However, 62.6% (*n* = 287) participants, on the other hand, had middle-to-high family incomes (10,000–30,000 SAR or more per month). Furthermore, only 33.6% (*n* = 154) of participants had medical insurance, and more than half did not have children 57.1% (*n* = 262). Overall statistics are explained in Table 1.

In this observational cross-sectional study, the most common source of information was also assessed among study participants (Table 2). Only 24.2% (*n* = 111) have watched national health shows on the TV, and 20.0% (*n* = 92) have listened to national medical programs on the radio in the last year, while only 17.2% (79) have watched or listened to international medical shows on the TV or radio on a regular basis. The internet was the most used source for cancer knowledge, 75.2% (*n* = 345), and was the second most preferred source for knowledge after health care workers, 49.7% and 50.1%, respectively. Cancer patients were the third most preferred group to obtain cancer information from. The radio, 46% (*n* = 211), and newspapers, 44% (*n* = 202), were the least favorite sources for obtaining cancer information. Other options such as books, brochures, flyers, family, friends, neighbors, television, lectures, and smartphone messages were preferred to some extent. About 36.6% (*n* = 168) of participants did not face any obstacles when searching for cancer-related information, while 32.9% (*n* = 151) had concerns about the reliability of the information present online. A small percentage of participants responded that they felt frustration (4.4%) and difficulty interpreting the meaning of statistical data (5.4%). The inclusion of cancer-related subjects in children’s educational curriculum was believed to be the best way to educate children about cancer, 48.4% (*n* = 222), while 20.5% (*n* = 94) of participants opposed educating children about cancer. On the other hand, 46.4% (*n* = 213) of participants suggested using the internet to educate adults about cancer, while 14.6% (*n* = 67) and 12.2% (*n* = 56) of participants supported the use of the educational curriculum and lectures for obtaining cancer knowledge, respectively.

Awareness of cancer causes and misconceptions were also assessed for all participants (Table 3). The relative knowledge among participants was good (≥50% positive answers). Causative agents were thought to be: irradiation exposure, 71.9% (*n* = 330), nuclear waste exposure, 80.0% (*n* = 367), air pollution, 51.9% (*n* = 238), cigarette smoking, 91.5% (*n* = 420), negative smoking, 78.9% (*n* = 362), hookah smoking, 85.6% (*n* = 393), bad dietary habits, 57.5% (*n* = 264), alcoholism, 74.7% (*n* = 343), pesticides, 61.4% (*n* = 282), chemicals, i.e., food additives and preservatives, 72.3% (*n* = 332), contact with cancer patients, 4.6% (*n* = 21). Other factors were known to some extent (50–40% positive response): X-ray exposure, 44.9% (*n* = 206), exposure to chemotherapeutics, 44.4% (*n* = 204), some drugs, 45.3% (*n* = 208), water contamination, 49.2% (*n* = 226), obesity, 40.7% (*n* = 187), lack of muscular exercise, 42.3% (*n* = 194), cleaning supplies, 43.6% (*n* = 200), paints and thinners, 44.2% (*n* = 203), psychiatric stress, 45.6% (*n* = 209).

There was poor knowledge about the risks of contraceptive pills, 16.3% (*n* = 75), sun exposure, 37.5% (*n* = 172), hair dyes, 27% (*n* = 126), viruses, 32.5% (*n* = 149), microwave, 16.8% (*n* = 77), cellular phones, 16.3% (*n* = 75). Lastly, 44.9% (*n* = 206) believed that envy or spiritual causes impose a risk for cancer.

Almost 70% of participants considered cancer a major health issue in Saudi Arabia. Cancer is a major issue in Saudi Arabia, according to participants, because of its increasing incidence, 44.0% (*n* = 202), incurability, 13.1% (*n* = 60), and lethal nature, 8.7% (*n* = 40). However, >60% (*n* = 276) believed that cancer is treatable. Almost 12% (54) of participants thought that surgery to eradicate cancer causes malignancy to spread to other body parts. Only 30% (*n* = 138) of the participants agreed that cancer in Saudi Arabia is associated with social stigma. Approximately half of the participants (*n* = 220) believed that Saudi patients lose hope after being diagnosed with cancer, and more than 54% (*n* = 253) of participants associated cancer with death. Study participants thought it is essential to support cancer patients psychologically, 94.2% (*n* = 432), and socially, 94.8% (*n* = 435). Furthermore, 64.5% (*n* = 296) thought cancer patients should be informed about their diagnosis, and >90% (*n* = 422) agreed that physicians and nurses should be compassionate with terminally ill cancer patients (Table 4).

The associations between the sociodemographic variables and the level of awareness are shown in Table 5. The factors that were statistically associated with a higher level of awareness included: older age (*p*-value < 0.032, 95% CI = −0.247–−0.011); high educational level (*p*-value < 0.018, 95% CI = 0.043–0.469); non-smokers (*p*-value—0.010, 95% CI = 0.172–1.269); parents (*p*-value—0.027, 95% CI = −0.685–−0.040); those who do not who have insurance (*p*-value—0.046, 95% CI = −0.006–−0.681).

## 4. Discussion

Awareness of cancer and carcinogens is considered a serious and important issue that affects public recognition, timely diagnosis, and prevention of the disease, especially with the increased number of cancer cases being reported in the last couple of decades in Saudi Arabia (136%) [6] and the lifestyle changes noticed in the country [3]. This highlights the need to assess the awareness, beliefs, and misconceptions about cancer and carcinogens. This study shows that most Saudi women understand that cancer is a significant and preventable health concern. Approximately half of the participants acknowledged the increasing incidence of cancer. Participants are also aware of the importance of psychological and social support for cancer patients.

The internet was the most used platform for obtaining cancer-related information. This finding is supported by (Algamdi et al.) [11] and (Ibrahim et al.) [12] who stated that the internet and social media are the most used sources for information. However, in contrast to (Alghamdi et al.) [11], our study found internet users to have the poorest knowledge about cancer among participants. Hence, social media platforms could be targeted for educating the public about cancer risks.

Regarding risk factors and carcinogens, respondents were aware of certain environmental and chemical carcinogens, most notably smoking. Many national (Sabi et al.) [13] (Alghamdi et al.) [11] (Ibrahim et al.) [12] (Ravichandran et al.) [14] and regional studies from the UAE (Ahmed et al.) [15], Oman (Alazri et al.) [16] and Lebanon (Kabalan) [17] showed that smoking was the most well-known risk factor. As (Alazri et al.) stated, this might be because smoking is culturally and religiously unacceptable in Muslim Gulf Arabian cultures. In addition, smoking is the most campaigned risk factor.

Radiation, contamination, and most chemical exposures were well-known factors too. However, when it comes to physical carcinogens, participants showed low awareness, with less than half attributing cancer to obesity and lack of physical activity, as well as poor knowledge about the risk of contraceptive pills, oncogenic viruses, and hair dyes. These results are concerning, given the higher tendency of obesity (Memish et al.) [18] and inactivity in Saudi females compared to Saudi males (Alhazza, A.) [19]. Another misconception noticed from the questionnaire was that approximately half of the respondents believed that envy or spiritual causes imposed a risk for cancer.

When comparing our study with similar observational studies, both regional and worldwide, the results were similar in almost all risk factors for cancer but varied in the knowledge of the risk of physical inactivity [16,20]. A study conducted in Riyadh showed similar knowledge about the risk of smoking and environmental carcinogens, which also noted poor knowledge about physical activity and obesity [13]. This was also supported by regional studies [16], which reported a similar pattern of awareness in Oman, with smoking being the most recognized risk factor, while only 30% knew the risk associated with physical inactivity [16]. In contrast, obesity and a lack of physical activity are well-known physical carcinogens among adults in Australia (more than 80%) [20].

Age was significantly associated with knowledge about cancer. Older women were more aware of cancer risk factors than younger women. This finding opposes (Ravichandran et al.) and (Ibrahim et al.), whose studies associated younger participants with better knowledge about cancer. Meanwhile, (Sabi et al.) concluded that age was not associated with cancer knowledge and attributed that to the availability of information to all ages through the internet. Higher education level was significantly associated with better cancer awareness. This is supported by (Ravichandran et al.) and (Alazri et al.) and contrasted by (Alghamdi et al.) who interestingly found that participants with higher education exhibited poorer knowledge.

This study was faced by few limitations. Recruitment was performed through convenient sampling by publishing the survey through social media channels, which could limit the generalizability of the results.

This study provides an insight into the level of cancer awareness among Saudi women and possible areas of improvement. In addition, it showed the internet as the preferred method of information delivery. This study could be used as a reference and a roadmap for awareness campaign programs and future national studies to improve the level of awareness and to strengthen screening programs.

## 5. Conclusions

The increasing number of cancer cases and the massive burden of cancer on health, society, and healthcare resources make awareness of cancer and carcinogens very important. There was a good level of knowledge among participants about cancer risk factors and environmental carcinogens. These results could help build awareness campaigns and educational programs based on the current knowledge, by targeting less known risk factors for cancer such as oncogenic viruses, physical activity, and obesity. In addition, educational material is best tangible through the internet and social media.

## Figures and Tables

**Table 1:** Demographics Data.

	Frequency (*n*)	Percentage (%)
**Region of Residence**		
Riyadh	316	68.8
Central region (other than Riyadh)	25	5.4
South region	15	3.3
East region	29	6.3
North region	14	3.1
West region	60	13.1
**Age**		
<18	13	2.8
18–24	149	32.5
25–34	135	29.4
35–44	83	18.1
45–54	50	10.9
55–64	29	6.3
**Education**		
Primary	3	0.7
Middle	13	2.8
High School	77	16.8
Bachelor	327	71.2
Master	39	8.5
**Smoking**		
No	416	90.6
Yes	43	9.4
**Marital status**		
Widowed	6	1.3
Single	225	49.0
Married	217	47.3
Divorced	11	2.4
**Employed**		
No	282	61.4
Yes	177	38.6
**Family income**		
<5000	52	11.3
10,000–20,000	173	37.7
5000–10,000	120	26.1
20,000–30,000	70	15.3
>3000	44	9.6
**Children**		
No	262	57.1
Yes	197	42.9
**Medical Insurance**		
No	305	66.4
Yes	154	33.6

**Table 2:** Source of information.

	Frequency		Percent	
**Did you watch any health or medical shows on TV in the past 12 months?**				
Don’t know	286		62.3	
Yes for national programs	111		24.2	
Yes for international programs	62		13.5	
**How many times have you watched national/international health programs in the last 12 months?**				
Once a week	72		15.7	
More than once a week	61		13.3	
Others	59		12.9	
I don’t know	267		58.2	
**Have you listened to any health/medical program from Radio in the last 12 months?**				
I don’t know	350		76.3	
Yes for national programs	92		20.0	
Yes for international programs	17		3.7	
**How many times have you listened to national/international health programs in the last 12 months?**				
Once a week	56		12.2	
More than once a week	34		7.4	
Others	48		10.5	
I don’t know	321		69.9	
**Have you search about cancer before?**				
no	138		30.1	
yes	321		69.9	
**The last time you seek cancer knowledge, what was your priority portal?**				
Family/Parents	2		0.4	
Friends/Relatives	10		2.2	
Internet	345		75.2	
TV	2		0.4	
Books	23		5.0	
Brochures/Flyers	4		0.9	
Cancer patient	4		0.9	
Health giver	1		0.2	
Healthcare Providers	28		6.1	
Social Media	23		5.0	
Others	17		3.7	
**What are the obstacles you faced during your cancer informatics search?**				
Heavy effort required	49		10.7	
Numerical and statistical jargons	14		3.1	
Frustration	20		4.4	
Difficult meanings	25		5.4	
Reliability for the informatics	151		32.9	
Others	32		7.0	
I did not meet obstacles	168		36.6	
**Best way, in your opinion, to educate children about cancer is:**				
Radio	1		0.2	
Internet	53		11.5	
TV	37		8.1	
The Educational Curriculum	222		48.4	
Lectures	18		3.9	
Brochures/Flyers	12		2.6	
Others	22		4.8	
Disagree to educate cancer to children	94		20.5	
**Best way in your opinion to orient adults about cancer is:**				
Radio	5		1.1	
Internet	213		46.4	
TV	68		14.8	
The Educational Curriculum	67		14.6	
Lectures	56		12.2	
Brochures/Flyers	32		7.0	
Others	18		3.9	
**Which of these sources you prefer to use to know about cancer?**			***n*** **(%)**	
	To some extent	Little	To a large degree	Absolutely No
**Books**	130 (28.3)	107 (23.3)	53 (11.5)	169 (36.8)
**Brochures and Flyers**	149 (32.5)	111 (24.2)	69 (15.0)	130 (28.3)
**Family, friends, or neighbors**	121 (26.4)	108 (23.5)	76 (16.6)	154 (33.6)
**Healthcare workers**	109 (23.7)	83 (18.1)	230 (50.1)	37 (8.1)
**Newspapers**	97 (21.1)	132 (28.8)	28 (6.1)	202 (44.0)
**Internet**	125 (27.2)	69 (15.0)	228 (49.7)	37 (8.1)
**a Cancer patient**	111 (24.2)	75 (16.3)	196 (42.7)	77 (16.8)
**Radio**	99 (21.6)	120 (26.1)	29 (6.3)	211 (46.0)
**TV**	124 (27.0)	127 (27.7)	61 (13.3)	147 (32.0)
**Lectures**	116 (25.3)	104 (22.7)	94 (20.5)	145 (31.6)
**Smart Phone messages**	102 (22.2)	106 (23.1)	80 (17.4)	171 (37.3)

**Table 3:** Awareness of factors related to cancer.

Do You Think the Following Is a Risk Factor for Cancer? *n* (%)			
	No	Do Not Know	Yes
**Irradiation Exposure**	39 (8.5)	90 (19.6)	330 (71.9)
**X-ray exposure**	105 (22.9)	148 (32.2)	206 (44.9)
**Nuclear Waste exposure**	19 (4.1)	73 (15.9)	367 (80.0)
**Exposure to Chemotherapeutics**	130 (28.3)	125 (27.2)	204 (44.4)
**Contraceptive pills**	212 (46.2)	172 (37.5)	75 (16.3)
**Some Drugs**	85 (18.5)	166 (36.2)	208 (45.3)
**Sun Exposure**	188 (41.2)	98 (21.4)	172 (37.5)
**Air Pollution**	106 (23.1)	115 (25.1)	238 (51.9)
**Water Contamination**	92 (20.0)	141 (30.7)	226 (49.2)
**Smoking**	92 (20.0)	141 (30.7)	226 (49.2)
**Cigarette smoking**	22 (4.8)	17 (3.7)	420 (91.5)
**Negative Smoking**	34 (7.4)	63 (13.7)	362 (78.9)
**Hookah smoking (shisha)**	30 (6.5)	36 (7.8)	393 (85.6)
**Bad dietary habits**	85 (18.5)	110 (24.0)	264 (57.5)
**Obesity**	131 (28.5)	141 (30.7)	187 (40.7)
**Lack of Muscular Exercise**	129 (28.1)	136 (29.6)	194 (42.3)
**Alcoholism**	43 (9.4)	73 (15.9)	343 (74.7)
**Pesticides**	61 (13.3)	116 (25.3)	282 (61.4)
**Cleaning Supplies**	102 (22.2)	157 (34.2)	200 (43.6)
**Paints, thinner and chemicals in Mechanics**	88 (19.2)	168 (36.6)	203 (44.2)
**Hair dyes**	146 (31.8)	187 (40.7)	126 (27.5)
**Psychological Stress**	122 (26.6)	128 (27.9)	209 (45.5)
**Viruses**	149 (32.5)	161 (35.1)	149 (32.5)
**Microwave**	246 (53.6)	136 (29.6)	77 (16.8)
**Food additives and preservatives**	31 (6.8)	96 (20.9)	332 (72.3)
**Cellular phones**	235 (51.2)	149 (32.5)	75 (16.3)
**Contact with Cancer patients**	395 (86.1)	43 (9.4)	21 (4.6)
**Envy and spiritual causes**	141 (30.7)	112 (24.4)	206 (44.9)

**Table 4:** Considering cancer as a health problem in Saudi.

		Frequency	Percentage		
**Do you think that cancer disease is a big health challenge in Saudi Arabia?**					
no		57	12.4		
Do not know		83	18.1		
yes		319	69.5		
**What is the main reason—in your opinion—for considering cancer a big problem in Saudi Arabia?**					
Increase in cancer incidence		202	44.0		
Psychological stress.		19	4.1		
Social conflicts		3	0.7		
Lack of services and personnel		13	2.8		
Lack of knowledge		29	6.3		
Cancer is painful		20	4.4		
Cancer is a deadly disease		40	8.7		
cost of therapy		10	2.2		
Cancer is incurable		60	13.1		
Cancer handicapping		3	0.7		
Others		5	1.1		
I do not know.		55	12.0		
**Do you agree with the following statement? *n* (%)**					
	Disagree	Strongly disagree	Agree	Strongly agree	Don’t know
**Can cancer be treated?**	8 (1.7)	2 (0.4)	276 (60.1)	69 (15.0)	104 (22.7)
**Is it better to inform cancer patient that he got cancer**	16 (3.5)	8 (1.7)	296 (64.5)	99 (21.6)	40 (8.7)
**Can surgery to eradicate cancer, causes spread of cancer to other body parts?**	110 (24.0)	73 (15.9)	44 (9.6)	10 (2.2)	222 (48.4)
**Do you think that Cancer in Saudi Arabia is associated with social stigma?**	49 (10.7)	52 (11.3)	109 (23.7)	29 (6.3)	220 (47.9)
**Do you think that the Saudi patient loses hope if he discovered his cancer?**	90 (19.6)	29 (6.3)	178 (38.8)	50 (10.9)	112 (24.4)
**Do you bind between cancer and death?**	99 (21.6)	38 (8.3)	199 (43.4)	54 (11.8)	69 (15.0)
**Is it important to support cancer patients psychologically?**	4 (0.9)	2 (0.4)	194 (42.3)	238 (51.9)	21 (4.6)
**Is it important to support cancer patients socially?**	0	3 (0.7)	191 (41.6)	244 (53.2)	21 (4.6)
**Is it a must for physicians to be compassionate with terminally ill cancer patient family?**	2 (0.4)	2 (0.4)	198 (43.1)	223 (48.6)	34 (7.4)
**Is it a must for nursing staff to be compassionate with terminally ill cancer patient family?**	1 (0.2)	2 (0.4)	200 (43.6)	222 (48.4)	34 (7.4)
**Is cancer treatment costly in Saudi Arabia?**	25 (5.4)	14 (3.1)	137 (29.8)	98 (21.4)	185 (40.3)

**Table 5:** Associations of level of knowledge with sociodemographic variables.

	Estimate	Std. Error	Wald	Sig.	95% Confidence Interval	
					Lower Bound	Upper Bound
City	0.055	0.047	1.354	0.245	−0.038	0.148
Age	−0.129	0.060	4.591	0.032	−0.247	−0.011
Educational level	0.256	0.109	5.557	0.018	0.043	0.469
Smoker	0.721	0.280	6.638	0.010	0.172	1.269
Marital status	−0.235	0.143	2.719	0.099	−0.515	0.044
Employment	0.309	0.167	3.435	0.064	−0.018	0.636
Family income	0.032	0.060	0.277	0.598	−0.087	0.150
Having children	−0.362	0.164	4.865	0.027	−0.685	−0.040
Having Medical insurance	0.344	0.172	3.991	0.046	0.006	0.681
Preferred way of learning	−0.001	0.056	0.000	0.992	−0.110	0.108

## Data Availability

The data used to support the findings of study can be available at reasonable request to the corresponding author.
